# Expression and processing analyses of wild type and p.R47H TREM2 variant in Alzheimer’s disease brains

**DOI:** 10.1186/s13024-016-0137-9

**Published:** 2016-11-25

**Authors:** Li Ma, Mariet Allen, Nobutaka Sakae, Nilufer Ertekin-Taner, Neill R. Graff-Radford, Dennis W. Dickson, Steven G. Younkin, Daniel Sevlever

**Affiliations:** 1 Department of Neuroscience, Mayo Clinic, Jacksonville, 32224 FL USA; 2 Department of Neurology, Mayo Clinic, Jacksonville, 32224 FL USA

**Keywords:** AD, TREM2, R47H, Microglia

## Abstract

**Background:**

Genetic analyses showed that the triggering receptor expressed in myeloid cells 2 (TREM2) p.R47H variant increases the risk for Alzheimer’s disease (AD). The question of whether the p.R47H mutation affects expression or function of the receptor remains unanswered. To address this question we quantified mRNA and analyzed protein profiles of WT and p.R47H TREM2 in human brains.

**Methods:**

Quantitative real-time PCR (qPCR) was performed using 2 sets of primers one that detects all *TREM2* mRNA isoforms and one specific for the alternative spliced isoform (*TREM2alt*) that encodes for the extracellular domain (soluble TREM2). Because in the brain TREM2 is expressed primarily in microglial cells, we also assessed the levels of *IBA1* to control for microglial variability across samples. For TREM2 protein quantitation and N-glycosylation processing, RIPA brain extracts were analyzed by Western blot before and after EndoH and PNGaseF treatments.

**Results:**

We identified statistically significant increased levels of *TREM2* transcripts in the temporal cortex of AD subjects when compared with controls; *TREM2alt* was likewise higher in AD cases, but was not significant after adjustment for covariates. Quantitative analysis of TREM2 protein confirmed qPCR results that showed higher levels in AD than in control brains. Among AD subjects, we observed a trend towards higher mRNA and protein TREM2 levels in carriers of the p.R47H risk allele. Analysis of individual TREM2 species found no difference in the relative amounts of mature and immature species, and carboxyl terminal fragments between non carriers and p.R47H samples. Furthermore, TREM2 species from either non carriers or p.R47H brains were equally susceptible to EndoH and PNGaseF treatments.

**Conclusions:**

Our results suggest that TREM2 expression is increased in AD. Furthermore, we provide evidence indicating that p.R47H mutation does not affect the levels of TREM2 either directly by altering expression or indirectly by affecting processing of the protein. Our data support previous findings that suggest that p.R47H variant affects TREM2 function by altering binding properties of the receptor rather than expression.

## Introduction

Alzheimer’s disease (AD) is the leading cause of dementia in the elderly. AD pathology is characterized by extracellular plaques composed primarily of amyloid beta protein (Aβ) and intracellular neurofibrillary tangles composed primarily of hyperphosphorylated tau. According to the amyloid cascade hypothesis, neurofibrillary tangles and the neuroinflammation invariably observed in AD are downstream events caused by Aβ accumulation [1]. There is mounting evidence, however, that the innate immune system plays an important role in AD. Support for involvement of innate immunity came from genetic and integrated system studies as follows. i) Network-based integrative analysis identified an immune/microglia module as one of the molecular systems most strongly associated with the pathophysiology of late onset AD [2]. ii) Genome-wide association studies identified genes of the immune system in general (CLU and MS4A) and of microglia function in particular (CD33 and CR1), in or close to the risk loci for late onset AD [3, 4]. iii) Heterozygous variants in triggering receptor expressed in myeloid cells 2 (TREM2), which encodes for a microglia receptor, associate with increased risk for AD. [5, 6]. Among these rare variants, p.R47H is the most commonly found associated with AD.

TREM2 belongs to the TREM family of receptors whose members are cell surface glycoproteins possessing an immunoglobulin-like extracellular domain, a transmembrane region, and a short cytoplasmic tail. In the brain, TREM2 is involved in regulation of the microglia inflammatory response and phagocytosis of cell debris. The receptor relies entirely on the adaptor protein DAP12 (aka TYROBP) for intracellular signaling, and this partnership is absolutely required for efficient phagocytosis [7].

Loss of function for either TREM2 or DAP12 has clinical implications, resulting in polycystic lipomembranous osteodysplasia with sclerosing leukoencephalopathy (PLOSL), a rare and fatal disease also known as Nasu Hakola disease, characterized by bones cysts and late-onset dementia [8, 9]. Although, the mechanism behind the pathology in PLOSL is not known, one hypothesis is that lack of either TREM2 or DAP12 activity impairs the clearance of apoptotic neurons by microglia, leading to the accumulation of necrotic debris [10].

It was recently demonstrated that TREM2 undergoes a proteolytic cleavage that releases its extracellular domain, leaving a carboxy terminal fragment (CFT) attached to the membrane [11]. In addition to the membrane-bound form, a soluble form of TREM2 (sTREM2) is detectable in plasma and CSF [12]. It is assumed that sTREM2 originates by the release of the receptor extracellular domain; however, in human brains the presence of an alternatively spliced *TREM2* transcript (*TREM2 alt*) encoding for sTREM2 has been reported [13].

In this study we used autopsy brains to quantitate *TREM2* and *TREM2alt* transcript levels in two cohorts: one composed of AD and normal control subjects and the other of p.R47H carriers and non-carriers (wild type). We also analyzed TREM2 at the protein level in p.R47H carriers, and compare its N-glycosylation profile with wild type brains.

## Findings

### Quantitative analysis of *TREM2* transcripts in control and AD brains

**Fig. 1:**
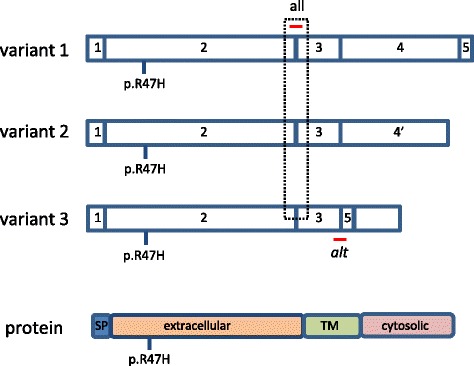
Localization of TaqMan probes for TREM2 transcripts. The cartoon illustrates 3 TREM2 transcripts with their exons. Variant 3 referred in the text as TREM2alt is devoid of exon 4. The TaqMan probe to detect all TREM2 transcripts spans from exon 2 to exon 3, and the probe to detect TREM2alt (alt) spans from exon 3 to exon 5. In the bottom of the Figure there is a representation of TREM2 protein with its different domains. The position of the p.R47H variant in the transcripts and protein is indicated. SP: signal peptide. TM: transmembrane domain

There are at least three TREM2 transcripts that are expressed in human brain [13]. Variant 1, the longest *TREM2* transcript consists of 5 exons, while variant 3 (the shortest) is an alternatively spliced isoform that excludes exon 4 (Fig. 1). Exon 4 contains the sequence for the TREM2 transmembrane domain; thus, variant 3 that was recently detected in human brains [13], likely encodes for a soluble form of the receptor (sTREM2). Previous studies using transfected cells suggest that proteolytic cleavage of the extracellular domain is the main mechanism by which sTREM2 is generated [11, 14]. Regardless of the mechanism, the potential relevance of sTREM2 as biomarker to monitor AD progression is highlighted by its elevation in the CSF of AD patients [15, 16].

In principle, the elevation of sTREM2 in AD patients could be the result of an overall increase of TREM2 expression and/or a specific increase in sTREM2 production. To address these possibilities, we quantified levels of total TREM2 transcripts and the alternatively spliced variant 3 (*TREM2alt*) in the temporal cortex of normal control and AD from post-mortem brains.

**Fig. 2:**
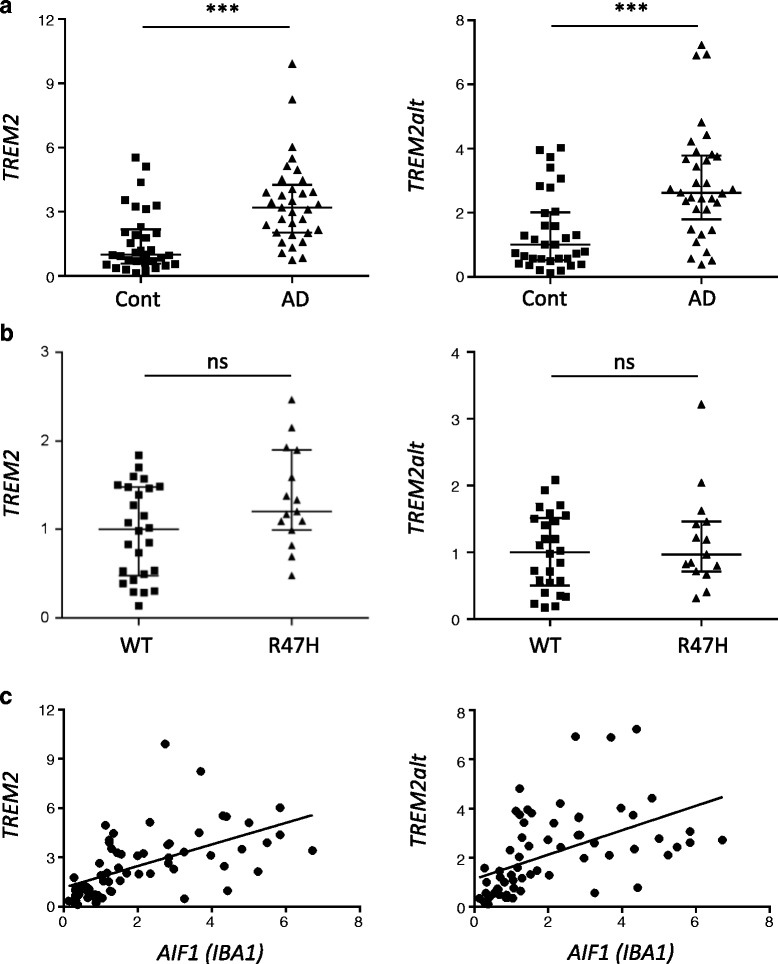
Expression of total and alternative spliced *TREM2* mRNA in AD and p.R47H carriers. Relative quantitation was performed using 2^-ΔΔCt^ (fold change) method. For each sample in the AD or p.R47H group the fold change (FC) value is expressed relative to the median value of TREM2 in control (**a**) or WT group (**b**) that was set at 1. The median FC value in AD samples is 3.19 for *TREM2* and 2.62 for *TREM2alt*; and in p.47H carriers is for *TREM2* 1.20 and 0.97 for *TREM2alt*. ***p < 0.0001. ns: not significant. **c**. Assessment of relative expression levels (2^-ΔΔCt^) for *TREM2* or *TREM2alt* indicates a positive association in all the samples (controls and ADs) for both *TREM2* transcripts with the microglial marker *AIF1* (*IBA1*) (R square values of 0.32 and 0.25 for *TREM2* and *TREM2alt*, respectively; and *p* < 0.0001 for both transcripts)

Demographics of the control and AD cases (cohort A) used for the analysis are summarized in Table 1. RNA was reversed transcribed and qPCR was performed using two sets of TaqMan primers: one that detects all *TREM2* mRNA variants and one specific for *TREM2alt*. Based on ΔCt calculations, the level of *TREM2alt* was between 5 and 7 times lower than of all *TREM2* transcripts combined. Relative expression levels were determined for all comparisons using the ΔΔCt method (2^-ΔΔCt^) and tested for differences between groups using the Wilcoxon rank sum test. We found statistically significant increased levels of *TREM2* transcripts in the temporal cortex of AD subjects when compared to controls (*p* = 1.14E-05, Table 2 and Fig. 2a). *TREM2alt* was also increased in AD (*p* = 7.28E-05, Table 2 and Fig. 2a) and the expression levels of *TREM2* and *TREM2alt* were correlated (r2 > 0.72, data not shown). The extent of the increase in AD brains was similar for both total *TREM2* and *TREM2alt* suggesting that the increase is not likely to be transcript-specific. To further assess significant results identified by the non-parametric Wilcoxon rank sum test (Table 2), we performed multivariable linear regression, using ΔCt as the expression variable, adjusting for known technical and biological covariates, and levels of the microglial marker *AIF1* (aka *IBA1*). Since we used ΔCt as the expression variable in this analysis, it should be noted that a negative beta indicates increased expression (Table 2). We confirmed the association of increased *TREM2* levels in the temporal cortex of AD compared to controls (*p* = 2.30E-02, Table 2) and although the increase for *TREM2alt* was not significant (*p* = 1.41E-01, Table 2), the association was in the same direction. This result indicates that *TREM2* levels are increased in AD even after taking into account the expression of the microglial marker *IBA1*; therefore, it is unlikely that the observed increase in *TREM2* expression is entirely due to increase number of microglial cells. Furthermore, we observed a positive correlation between *TREM2* and *TREM2alt* with *IBA1* expression levels in control and AD samples (Fig. 2c).

**Table 1:** Demographic of studied cases

	N	Mean age at death (SD)	Female: N (%)	Apoε4: N (%)	RIN: median (range)
AD^a^	33	72.7 (6.2)	17 (52%)	16 (48%)	6.0 (5.0–7.9)
CONT^a^	33	70.7 (5.7)	18 (55%)	15 (45%)	7.0 (5.3–8.4)
WT (CC)^b^	26	79.7 (8.5)	19 (73%)	20 (77%)	6.6 (5.5–8.6)
R47H (CT)^b^	15	81.5 (8.3)	11 (73%)	9 (69%)	6.3 (5.5–8.4)

^a^cohort A ^b^cohort B

**Table 2:** Summary of expression association analysis

		Wilcoxon rank sum (2^-ΔΔCt^)		Linear Regression (ΔCt)	
Dx	Transcript	Median (IQR)	*p*-value	Beta (se)	*p*-value
AD^a^	*TREM2*	3.19 (2.03–4.06)	1.14E-05	−0.51 (0.22)	2.30E–02
AD^a^	*TREM2Alt*	2.62 (2.10–3.75)	7.28E-05	−0.33 (0.22)	1.41E-01
AD (R47H)^b^	*TREM2*	1.20 (1.04–1.74)	7.61E-02		
AD (R47H)^b^	*TREM2Alt*	0.97 (0.75–1.44)	6.02E-01		

^a^cohort A ^b^cohort B

*TREM2* expression analysis in cerebellum (a brain region with minimal neuronal loss in AD) was confounded by the low levels of *TREM2* in that brain region; however, the same trend towards increased expression in AD compared to controls was observed (data not shown).

Similar analysis was performed with RNA from an independent cohort (B) of p.R47H TREM2 carriers (CT) and those homozygous for the major allele (CC) (referred also in the text and Figures as wild type). All p.R47H carriers had pathologically-confirmed typical AD, except for one sample that had posterior cortical atrophy (PCA), an AD subtype; wild type (WT) non carriers used for the analysis were also AD and one was PCA. The Wilcoxon-rank-sum test indicated higher *TREM2* and *TREM2alt* levels in carriers of the p.R47H risk allele; however, differences between the two groups were not statistically significant (Fig. 2b and Table 2).

### Quantitative analysis of TREM2 protein in control and AD brains

**Fig. 3:**
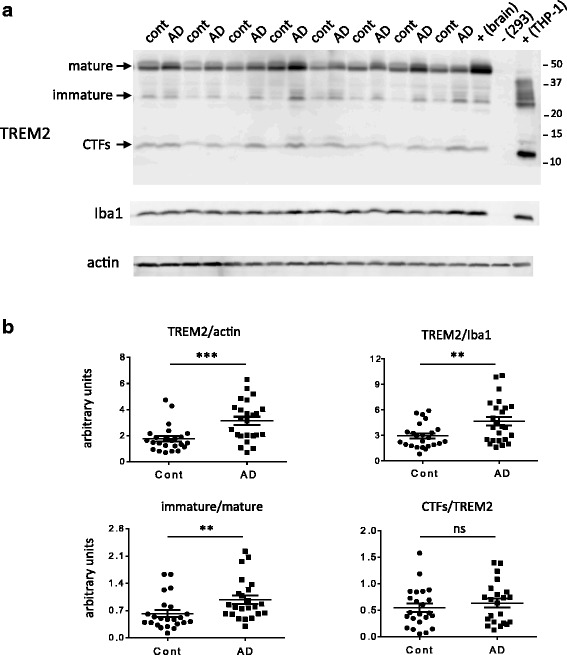
TREM2 protein expression in control and AD brains. Temporal cortices were extracted with RIPA buffer and 100 μg of the soluble fraction (supernatant of 20,000 g centrifugation for 30 min) was used for the analyses. **a**. Western blot of a representative gel probed with a C-terminal TREM2 antibody, *Iba1*, and actin antibodies. Three main TREM2 species are indicated: mature, immature and carboxy terminal fragment (CTF). To confirm the specificity of the C-terminal TREM2 antibody two positive and one negative controls were included. The positive (+) controls were lysates of a human brain and THP-1 cells (a human monocyte cell line) previously tested with B-3, a well characterized TREM2 antibody. The negative (−) control was a lysate of 293 cells that do not express endogenous TREM2. **b**. The signal intensity of all three species TREM2 species, Iba1, and actin in each sample was quantitated using ImageQuant software and used for plotting TREM2 (all species) normalized to actin and Iba1 levels, the ratio between immature and mature species, and CTF species normalized to TREM2 (full length: immature and mature species). ***p < 0.0001, **p < 0.001, and ns: not statistically significant

**Fig. 4:**
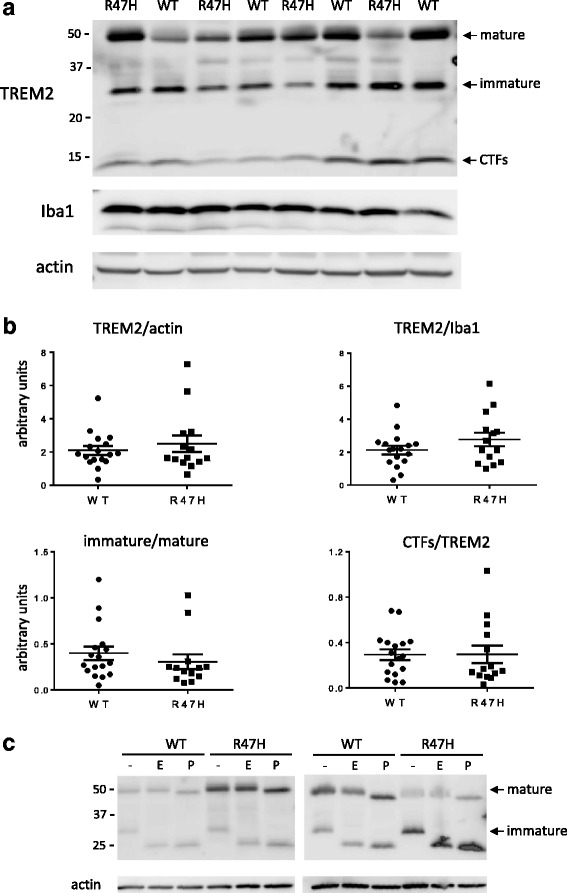
TREM2 expression and deglycosylation profiles in WT and p.R47H carriers. RIPA extracts from temporal cortices were used for the analyses. **a**. Western blot of a representative gel probed with C-terminal TREM2 antibody, Iba1, and actin antibodies. Three main TREM2 species are indicated: mature, immature and carboxy terminal fragment (CTF). **b**. The signal intensity in TREM2 species in WT and p.R47H samples (*N* = 16 for each group) was quantitated as previously described, and was plotted after normalization to actin and Iba1. The ratio between immature and mature TREM2 species and between CTF species normalized to TREM2 (full length: immature and mature species) were also plotted. **c**. Soluble fractions from RIPA buffer brain extracts were incubated in reaction buffers without enzymes (controls) or with endoH (1000U) or PNGaseF (1000U) overnight at 37 °C. The whole reaction mixture was analyzed by Western blot probed with a C-terminal TREM2 antibody. Blots containing two representative samples from WT and p.R47H brains are shown

Protein analysis was performed in the same brain samples in which TREM2 transcripts were previously evaluated. We performed quantitative Western blot to establish if the increase in *TREM2* mRNA in AD samples is reflected at the protein level. RIPA buffer extracts from temporal cortexes from a subset of control and AD samples (*N* = 48 out of 66) analyzed for TREM2 transcripts were used and the blots were probed with a monoclonal antibody against the carboxy terminus of TREM2. This antibody allowed the detection of three TREM2 species: mature (~50 kDa), immature (~30 kDa), and carboxy terminal fragment (CTF) (Fig. 3a). The CTF species were identified on Western blots based on its molecular weight and immunoreactivity with a C-terminal antibody (Fig. 3a), but not with a N-terminal antibody (data not shown). The designation as mature or immature TREM2 species was based on their resistance or susceptibility to EnodH and PNGaseF, respectively (Fig. 4c). Quantitation of all TREM2 species normalized to Iba1 reveals a statistically significant elevation in AD samples as compared to controls (Fig. 3b). To evaluate TREM2 processing we also compared the ratio between immature and mature species and the levels of CTF species between the two groups. The ratio between immature and mature TREM2 was significantly higher (*p* < 0.001) in the AD samples, but not significant differences were detected when the levels of CTF species between controls and ADs were compared (Fig. 3b).

As might be expected given the observed increase of both TREM2 RNA and protein in AD subjects when compared with controls, Spearman rank analysis indicated a positive correlation between TREM2 mRNA and protein values (rho = 0.36, *p* = 0.012) when assessed in the same subjects. Our data are in agreement with a previous study of postmortem human brains that reported higher TREM2 protein levels in AD than in controls [17].

### Expression and deglycosylation profiles of WT and p.R47H TREM2

To establish if the p.R47H mutation alters either the expression and/or glycosylation of TREM2, we subjected brain samples from WT and p.R47H carriers to quantitative Western blot analysis and enzymatic treatments with glycosidases. The distribution of TREM2 signal between mature, immature, and CTF species varied among samples (Fig. 4a), but statistical analysis shows no significant difference in total TREM2 expression between WT and p.R47H carriers (Fig. 4b). Furthermore, the ratio between immature and mature species is not altered by the presence of the mutation, suggesting that it does not affect intracellular trafficking of the receptor (Fig. 4b). We also looked at the impact of p.R47H on the extent of TREM2 proteolytic cleavage by quantitating CTF species relative to total TREM2 species in each sample and found no difference with WT subjects (Fig. 4b). This data indicates that the p.R47H mutation does not affect TREM2 delivery to the cell surface where CTF species are generated. The intracellular trafficking of TREM2 can be monitored by assessing modifications to its N-glycan chains using EndoH and PNGaseF enzymes. EndoH susceptible TREM2 species (immature) are still in the endoplasmic reticulum while resistant (mature) have left that compartment. All N-glycosylated species are susceptible to PNGaseF regardless of their intracellular localization. Removal of N-glycan chains from TREM2 by either EndoH or PNGaseF results in a lower molecular weight of the protein, a change that can be detected by Western blot. Figure 4c shows representative deglycosylation experiments of WT and p.R47H samples using EndoH and PNGaseF enzymes with different proportions of ~50 kDa and ~30 kDa TREM2 species. We consistently observed that in WT and p.R47H samples the ~50 kDa species (mature) was resistant to EndoH and susceptible to PNGaseF and that the ~30 kDa species (immature) was susceptible to both enzymes. The nature of the molecular weight difference between mature and immature TREM2 species is unknown; however based on the enzymatic deglycosylation experiments the molecular weight difference is not due to glycosylation (Fig. 4c).

Our results contrast with a recent study that found elevated mRNA but decreased protein expression in p.R47H carriers [18]. Our data indicate a trend, albeit not significant, towards both higher mRNA and protein TREM2 levels as a result of the mutation. Furthermore, our results do not support previous in vitro studies that suggested that p.R47H affects TREM2 intracellular trafficking [14, 19]. Rather, our data are more in line with a recent study showing higher TREM2 levels in CSF of p.R47H carriers than in controls [12].

In conclusion, we demonstrated that total *TREM2* including *TREM2alt* transcript and TREM2 protein levels are increased in AD. Our study indicates that proteolytic cleavage of the receptor is likely the main mechanism for sTREM2 generation in the brain based on the very low levels of expression of *TREM2alt* and the detection of CTF species in brain extracts. Finally, our data supports the notion that the p.R47H mutation does not affect processing (i.e., N-glycosylation and cell surface proteolytic cleavage) of TREM2.

## Material and methods

### Brains

In the autopsy-confirmed series, all brains were from the brain bank at Mayo Clinic in Jacksonville. The diagnosis of confirmed AD was made according to accepted neuropathological criteria. All late-onset AD brains analyzed had a Braak neurofibrillary tangle stage of IV or greater. Brains employed as controls had a Braak stage of less than III. Most had other brain pathology unrelated to AD and included cerebrovascular pathology, frontotemporal dementia, Lewy body diseases, corticobasal degeneration, argyrophilic grain disease, multi-system atrophy, amyotrophic lateral sclerosis, and progressive supranuclear palsy. No subjects in this study carried familial Alzheimer’s disease mutations in APP, PSEN or the TREM2 R47H mutation, unless indicated.

### qPCR

Total RNA was extracted from 50 mg of frozen temporal cortex and cerebellum of late-onset Alzheimer’s disease brains and controls with Trizol (Sigma). Purelink DNAse (Invitrogen) was used to remove genomic DNA, and RNA was reverse transcribed to single-stranded cDNA using the High-Capacity cDNA Archive Kit from AppliedBiosystems. Quantitative real-time PCR was performed in triplicate for each sample using ABI TaqMan Low Density expression Arrays (384-Well Micro Fluidic Cards) with pre-validated TaqMan Gene Expression Assays. Glyceraldehyde-3-phosphate dehydrogenase (GAPDH, Hs02758991_g1) and hypoxanthine phosphoribosyltransferase 1 (HPRT1, Hs02800695_m1) were used as housekeeping genes. The following additional TaqMan Gene Expression assays were used: Hs00219132_m1 to capture all TREM2 transcripts, a custom assay was designed for *TREM2alt* (assay ID:AI89K96), and Hs00610419_g1 for *AIF1* (aka *IBA1*). Real-time PCR cycle threshold (CT) raw data was collected and exported using the ABI PRISMH SDS software version 2.2. The variable ΔCT denotes the difference between the averaged CT values for the *TREM2* transcripts and that for the housekeeping genes. The fold change (2^-ΔΔCt^) for each sample was calculated relative to the median ΔCT in control samples (AD vs control) or subjects homozygous for the major allele (R47H), as relevant.

### EndoH and PNGaseF treatments

100 μg of RIPA-soluble fractions were boiled for 5 min in denaturing buffer. After cooling, the samples were incubated with 2 μl of either EndoH (1000U) or PNGaseF (1000U) overnight at 37 °C in their respective assay buffers supplemented with 1% NP-40. Both enzymes and buffers were from New England Biolabs. The whole reaction was loaded on a gel for Western blot analysis.

### Western blot

Temporal cortexes were extracted with RIPA buffer (Sigma) and soluble fractions were generated by centrifugation for 30 min at 20,000 g. Protein concentration in the RIPA-soluble fractions was determined by BCA assay and 100 μg from each sample were run on 4–20% gradient Tris-glycine Novex gels (Invitrogen). Transfer of the proteins to nitrocellulose membranes was carried out at 70 V for 2 h. The membranes were then blocked for 1 h with 5% milk in TBS 0.05% Tween-20 and incubated overnight with the following TREM2 antibodies: a C-terminal Ab D8I4C (1–500, Cell Signaling) and N-terminal Ab 175262 (1–500, Abcam). The other primaries antibodies used were actin (I-19 1–100, Santa Cruz) and Iba1 (1–2000, Abcam 178847). The blots were washed five times with TBS 0.05% Twenn-20 and incubated with an anti-rabbit HRP antibody (1–2000) for 1 h at room temperature. The blots were developed with SuperSignal West Femto reagent (Pierce), imaged with the Fujifilm Luminescent Image Analyzer LAS4000 System, and the bands were quantitated using ImageQuant software.

### Statistical analysis

Relative expression levels were calculated using the 2-^ΔΔCt^ method, and tested for differences between groups using the Wilcoxon rank sum test with Prism software. When this test was significant, we used multi-variable linear regression to further assess the association, using ΔCt as the expression variable and adjusting for the following covariates: age at death (years), sex, RNA integrity number (RIN), qPCR plate and measured levels (ΔCt) of *IBA1*. Statistical analyses were performed using the R Statistical Software (R Foundation for Statistical Computing, version 3.2.3). Prism software was used for statistical analysis of data presented in Figs. 3 and 4.

## Abbreviations

AD: Alzheimer’s disease
Aβ: Amyloid beta
CFT: Carboxy terminal fragment
Ct: Cycle threshold
qPCR: Quantitative real time polymerase chain reaction
sTREM2: Soluble TREM2
TREM2: Triggering receptor expressed in myeloid cells 2
*TREM2alt*: Alternative spliced TREM2

## References

[1] Hardy J, Selkoe DJ (2002). The amyloid hypothesis of Alzheimer’s disease: progress and problems on the road to therapeutics. Science.

[2] Zhang B, Gaiteri C, Bodea L-G, Wang Z, McElwee J, Podtelezhnikov Alexei A (2013). Integrated systems approach identifies genetic nodes and networks in late-onset Alzheimer’s disease. Cell.

[3] Lambert J-C, Heath S, Even G, Campion D, Sleegers K, Hiltunen M (2009). Genome-wide association study identifies variants at CLU and CR1 associated with Alzheimer’s disease. Nat Genet.

[4] Naj AC, Jun G, Beecham GW, Wang L-S, Vardarajan BN, Buros J (2011). Common variants at MS4A4/MS4A6E, CD2AP, CD33 and EPHA1 are associated with late-onset Alzheimer’s disease. Nat Genet.

[5] Guerreiro R, Wojtas A, Bras J, Carrasquillo M, Rogaeva E, Majounie E (2013). TREM2 variants in Alzheimer’s disease. N Engl J Med.

[6] Jonsson T, Stefansson H, Steinberg S, Jonsdottir I, Jonsson PV, Snaedal J (2013). Variant of TREM2 associated with the risk of Alzheimer’s disease. N Engl J Med.

[7] Klesney-Tait J, Turnbull IR, Colonna M (2006). The TREM receptor family and signal integration. Nat Immunol.

[8] Paloneva J, Kestila M, Wu J, Salminen A, Bohling T, Ruotsalainen V (2000). Loss-of-function mutations in TYROBP (DAP12) result in a presenile dementia with bone cysts. Nat Genet.

[9] Paloneva J, Mandelin J, Kiialainen A, Bohling T, Prudlo J, Hakola P (2003). DAP12/TREM2 deficiency results in impaired osteoclast differentiation and osteoporotic features. J Exp Med.

[10] Thrash JC, Torbett BE, Carson MJ (2009). Developmental regulation of TREM2 and DAP12 expression in the murine CNS: implications for Nasu-Hakola disease. Neurochem Res.

[11] Wunderlich P, Glebov K, Kemmerling N, Tien NT, Neumann H, Walter J (2013). Sequential proteolytic processing of the triggering receptor expressed on myeloid cells-2 (TREM2) protein by ectodomain shedding and gamma-secretase-dependent intramembranous cleavage. J Biol Chem.

[12] Piccio L, Buonsanti C, Cella M, Tassi I, Schmidt RE, Fenoglio C (2008). Identification of soluble TREM-2 in the cerebrospinal fluid and its association with multiple sclerosis and CNS inflammation. Brain.

[13] Jin SC, Benitez BA, Karch CM, Cooper B, Skorupa T, Carrell D (2014). Coding variants in TREM2 increase risk for Alzheimer’s disease. Hum Mol Genet.

[14] Kleinberger G, Yamanishi Y, Suárez-Calvet M, Czirr E, Lohmann E, Cuyvers E (2014). TREM2 mutations implicated in neurodegeneration impair cell surface transport and phagocytosis. Sci Transl Med.

[15] Jin SC, Carrasquillo MM, Benitez BA, Skorupa T, Carrell D, Patel D (2015). TREM2 is associated with increased risk for Alzheimer’s disease in African Americans. Mol Neurodegener.

[16] Piccio L, Deming Y, Del-Aguila JL, Ghezzi L, Holtzman DM, Fagan AM (2016). Cerebrospinal fluid soluble TREM2 is higher in Alzheimer disease and associated with mutation status. Acta Neuropathol.

[17] Lue LF, Schmitz CT, Serrano G, Sue LI, Beach TG, Walker DG (2015). TREM2 protein expression changes correlate with Alzheimer’s disease neurodegenerative pathologies in post-mortem temporal cortices. Brain Pathol.

[18] Roussos P, Katsel P, Fam P, Tan W, Purohit DP, Haroutunian V (2015). The triggering receptor expressed on myeloid cells 2 (TREM2) is associated with enhanced inflammation, neuropathological lesions and increased risk for Alzheimer’s dementia. Alzheimers Dement.

[19] Park J-S, Ji IJ, An HJ, Kang M-J, Kang S-W, Kim D-H (2015). Disease-associated mutations of TREM2 Alter the processing of N-linked oligosaccharides in the golgi apparatus. Traffic.

